# A Tribute from Dr. Bogart

**Published:** 1914-06

**Authors:** G. Henri Bogart


### A Tribute from Dr. Bogart.

I shall not try to tell you how Dr. Daniel's death shocked me, even though in measure I was prepared for the announcement. It was never mine to clasp the hand of Dr. Daniel, nor to look into his eyes, and yet, from the first, I was strangely attracted to him, to that wonderful optimistic altruism that consecrated his life to the service—not alone willing, rather gladsome—for "Those others," for the uplift and betterment of humanity, for those who most need help and consolation. I loved him for his fearlessness, for his daring to be himself, and among the circle of friends in the broadest sense I always felt that Dr. Daniel was one of the chosen few, who had come into the inner circle.

"Many pause, clasp hands, and name you,
Some make pulses faster beat;
But that friendship, which shall claim you,
Is the pearl makes life replete."

These lines from one of my poems best express my thoughts, for there was a common bond long enough to reach across the void and strong enough to make as naught the distance.

While he and his work meant so much to me, it would be but seeking to paint the rose for me to say aught of that closer relationship that linked you with him and which entered so freely and so fully in accord with his spirit. He was my friend; more I could never hope to say, and whatever I may do to aid the careless world along the same lines as his endeavors will find added incentive from knowing him.

He lives; he will live, not in the narrow ideals of creeds, but in the deeds that he has done, for our influences touch others, and they sway yet others and so, on and on, until even our unspoken thoughts shall spread until they break upon the beach of the fartherest shore of Eternity.

He sleeps; yet his work and spirit are working onward and upward a benison to "Those others."

I would that I might say more, only words mean so little. I I trust that you may find gladness in the future; that you may have him ever with you in spirit.

Sincerely your friend,

G. Henri Bogart.

